# Integrating transdiagnostic and holistic approaches in early neurodevelopmental screening

**DOI:** 10.1111/dmcn.70096

**Published:** 2025-11-30

**Authors:** Kahoko Yasumitsu‐Lovell

**Affiliations:** ^1^ Science Research Center Kochi Japan

## Abstract

**This commentary is on the original article by Luke et al. on pages** 381–393 **of this issue.**

Early identification of neurodevelopmental problems is crucial to optimize developmental outcomes and enable timely intervention. However, conventional screening practices have often been condition‐specific and grounded in biomedical frameworks that insufficiently capture developmental diversity or cultural context. Luke et al. advance the field through a culturally co‐designed, transdiagnostic study involving Australian First Nations infants.^1^ Their use of detailed motor and neurological assessments—the Motor Optimality Score–Revised (MOS‐R) and the Hammersmith Infant Neurological Examination (HINE)—demonstrates the capacity of these tools to predict a spectrum of neurodevelopmental outcomes, including cerebral palsy, autism, and fetal alcohol spectrum disorder.

This study is significant for two reasons. First, it extends early detection beyond single‐diagnosis paradigms by adopting a transdiagnostic framework, recognizing that many neurodevelopmental disorders share overlapping early indicators across motor, cognitive, and behavioural domains. This approach reflects emerging evidence that early neurodevelopmental differences are dimensional rather than categorical, and that early identification should focus on functional support rather than diagnostic labelling.^2^ Second, the study exemplifies a culturally responsive model of care. Through co‐design with First Nations communities, training of local health workers, and adaptation of materials to local languages and worldviews, the authors achieved an 86% follow‐up rate—substantially higher than typical neonatal screening programmes. This illustrates that cultural safety is integral to scientific validity and essential for equitable engagement.

Several considerations arise from this work. The first point relates to the interpretation of transdiagnostic markers. While reduced MOS‐R and HINE scores indicate heightened developmental vulnerability, these findings should not be regarded as disorder‐specific in early infancy. Neurodevelopmental disorders frequently co‐occur, and early manifestations may evolve into different or multiple conditions over time; therefore, longitudinal clinical follow‐up is crucial. As the authors note, prioritizing sensitivity minimizes missed cases but increases false positives. In resource‐limited contexts—often the reality—excessive sensitivity may heighten parental anxiety or strain referral pathways. Accordingly, transdiagnostic screening should be implemented alongside staged follow‐up and clear, family‐centred communication strategies.

A second consideration concerns more holistic integration of assessment tools. Combining clinician‐administered measures such as the HINE and MOS‐R with caregiver‐reported tools can enhance ecological validity and capture developmental differences observable in everyday contexts. A screening tool like the ESSENCE‐Q (Early Symptomatic Syndromes Eliciting Neurodevelopmental Clinical Examinations Questionnaire) exemplifies this approach, as it includes other common manifestations of neurodevelopmental problems such as sleeping and feeding difficulties.^3^ Administered to guardians, clinicians, or researchers, this questionnaire encompasses communication, motor, sensory, and behavioural domains, and complements the clinician‐administered measures evaluated by Luke et al. Integrating such tools within early screening frameworks could strengthen both developmental breadth and cultural applicability.

Clinically, this work highlights that how screening is conducted is as important as what is screened. Embedding co‐designed, culturally informed programmes within local service structures can help ensure that early identification is equitable, trusted, and sustainable.^4^ The success of the Learning through Everyday Activities with Parents for infants with Cerebral Palsy (LEAP‐CP) model provides a valuable template for implementation in other culturally diverse or underserved populations, reinforcing that early developmental support must reflect both scientific evidence and community values.

Future research should extend these findings longitudinally to establish predictive validity for non‐cerebral palsy neurodevelopmental delay outcomes and refine culturally appropriate cut‐off scores. Comparative studies combining structured neurological assessments with brief holistic questionnaires could help define scalable models that integrate medical precision with family‐centred care.

By uniting robust methodology with cultural and contextual understanding, Luke et al. present a model for inclusive early detection that could ultimately inform treatment strategies.^5^ Their work affirms that the future of neurodevelopmental screening lies not only in diagnostic accuracy but in responsiveness—to culture, community, and the full spectrum of early development.

## Data Availability

Not required.
